# FSH receptor binding inhibitor up-regulates ARID1A and PTEN genes associated with ovarian cancers in mice

**DOI:** 10.1590/1414-431X20198381

**Published:** 2019-06-19

**Authors:** Zhuandi Gong, Xiaoyun Shen, Juan Yang, Kun Yang, Shengju Bai, Suocheng Wei

**Affiliations:** 1 Medicine College Hospital, Northwest Minzu University, Lanzhou, China; 2 State Engineering Technology Institute for Karst Desertification Control, Guizhou Normal University, Guiyang, China; 3 School of Life Science and Engineering, Southwest University of Science and Technology, Mianyang, China; 4 College of Life Science and Engineering, Northwest Minzu University, Lanzhou, China

**Keywords:** ARID1A, PTEN, FSH receptor binding inhibitor, Ovarian cancer

## Abstract

Experiments were conducted to determine if the follicle-stimulating hormone (FSH) receptor binding inhibitor (FRBI) impacts the expression levels of AT-rich interactive domain-containing protein 1A (ARID1A) and phosphatase and tensin homolog (PTEN) in ovaries and blood, as well as expressions of follicle-stimulating hormone cognate receptor (FSHR) gene and proteins. Mice in FRBI-10, FRBI-20, FRBI-30, and FRBI-40 groups were intramuscularly injected with 10, 20, 30, and 40 mg FRBI/kg, respectively, for five consecutive days. Western blotting and qRT-PCR were utilized to determine expression levels of ARID1A and PTEN proteins and mRNAs. Serum ARID1A and PTEN concentrations of the FRBI-40 group were higher than the control group (CG) and FSH group (P<0.05). FSHR mRNA levels of FRBI-20, FRBI-30, and FRBI-40 groups were lower than that of CG and FSH groups on day 15 (P<0.05 or P<0.01). Expression levels of FSHR proteins of FRBI-30 and FRBI-40 groups were lower than those of CG and FSH groups (P<0.05). Levels of ARID1A and PTEN proteins of the FRBI-30 group were greater than CG on days 20 and 30 (P<0.05). FRBI doses had significant positive correlations to levels of ARID1A and PTEN proteins. Additionally, ARID1A and PTEN had negative correlations to FSHR mRNAs and proteins. A high dose of FRBI could promote the expression levels of ARID1A and PTEN proteins in ovarian tissues. FRBI increased serum concentrations of ARID1A and PTEN. However, FRBI depressed expression levels of FSHR mRNAs and proteins in mouse ovaries.

## Introduction

Early diagnosis of ovarian cancers is a critical factor in improving the survival rate of patients. Several factors impact the carcinogenesis and prognosis (1) of ovarian cancer. The pathogenesis of ovarian cancer is closely related to ARID1A (AT-rich interactive domain-containing protein 1A), which is present in up to 57% of patients (2) and acts as a tumor suppressor in various cancers (3). Patients with ovarian clear cell carcinomas commonly respond poorly to standard platinum-based chemotherapy (4). Loss of ARID1A expression has also been associated with a shorter progression-free survival (5). Dysfunction of ARID1A can lead to abnormal chromatin remodeling, resulting in the carcinogenesis of ovarian and gynecologic cancers. Earlier studies reported that ARID1A mRNA expression was lower in endometriosis tissues than that in normal endometrial tissues. Low expression levels of ARID1A gene may contribute to the tumor's tendency to transform malignantly (6). Currently, growing evidence has indicated that ARID1A may have a widespread role in the suppression of various tumors. The comprehensive study of ARID1A gene can provide a basis for earlier diagnosis and effective molecular target therapy (7).

Phosphatase and tensin homolog (PTEN) is a novel anti-oncogene (8) that, as a tumor suppressor gene, plays important roles in suppressing cancer and regulating apoptosis, enhancing the sensitivity of cancer cells to anticancer agents (9). Expression of PTEN in ovarian cancer tissue is negatively associated with clinical stage and differentiation degree. Expression of PTEN mRNA was significantly downregulated in blood plasma of epithelial ovarian cancer (EOC) patients compared to the ovarian tumor patients (9). Therefore, the overall survival of patients with PTEN positive expression was significantly longer than that of those with PTEN negative expression (10).

Follicle-stimulating hormone (FSH) promotes the rapid growth and survival of EOC cells, and facilitates entry into the carcinogenesis process. FSH also inhibits apoptosis of ovarian cancer cells. FSH exerts its functions via binding to its cognate receptor (FSHR) expressed in ovarian cancers and gynecologic malignancies (1). FSHR overexpression may be associated with enhanced levels of potential oncogenic pathways and increased proliferation in EOC cells. Therefore, inhibition of FSHR overexpression may be beneficial for suppressing the carcinogenesis and progression of EOC. However, whether FSHR plays a role in ovarian cancer development is still uncertain (11).

FSH receptor binding inhibitor-8 (FRBI-8), a non-steroidal antagonist for FSH, was identified from sheep and human ovarian follicular fluid (12,13). FRBI not only blocked the binding of FSH to FSHR, but also altered FSH actions (14). Our initial study revealed that the addition of 10 to 40 μg/mL FRBI into *in vitro* maturation (IVM) medium reduced the maturation rate, enhanced the apoptosis rate, and decreased the proliferation capacity of sheep cumulus-oocyte complex. Additionally, FRBI decreased FHS concentrations and increased estradiol (E_2_) concentrations in the IVM medium (15). Currently, there is scarce information about effects of FRBI on the gene levels associated with ovarian cancers in humans and animals (16). Little is known about whether FRBI modulates ARID1A and PTEN levels in normal ovarian and cancerous tissues (17,18).

Based on the previous research, we hypothesized that FRBI impacts the expression levels and production of ARID1A, PTEN, and FSHR, which are associated with carcinogenesis and progression of ovarian cancer. The present study aimed to evaluate the effects of FRBI on the levels of ARID1A and PTEN genes in ovarian tissues and blood, to determine the regulating effects of FRBI on expressions of FSHR genes and proteins as well as phosphorylation of FSHR in the ovarian tissues. Additionally, we aimed to investigate the correlation between these factors, to further explore the signal pathway and molecular mechanism of FRBI actions. We expect to find a novel preventive and therapeutic agent for ovarian cancers.

## Material and Methods

### Animal treatment

FSH receptor binding inhibitor peptide (FRBI, an 8-peptide including H-Ter-Glu-Asn-Leu-Glu-Pro-Asn-Gly-Glu-Gly-NH_2_) of 99.9% purity was synthesized by Nanjing Peptide Biotech Co. Ltd., China (CAS: 163973-98-6) referring to initial reports (13) and characterized before using. FRBI solution was prepared according to the method established in our laboratory (19). The concentration of FRBI was 1 mg/mL.

One hundred and eighty pre-puberty Kunming female mice (*Mus musculus*) were purchased from Experiment Animal Center, Lanzhou University (License No. SCXK (Gansu) 2005-0007). Mice were utilized in experiments at 21 days of age and body weight of 22.3±1.52 g after they acclimatized for 10 days prior to experiments. All mice were randomized into FRBI, FSH, and control groups (CG) (n=30). Mice of the FRBI group were again divided into FRBI-10, FRBI-20, FRBI-30, and FRBI-40 groups. They were intramuscularly injected with FRBI at the doses of 10, 20, 30, and 40 mg/kg, respectively, once a day for five consecutive days. Mice in the FSH group were intramuscularly injected with 10 IU FSH once a day for five consecutive days, which was used as a positive control group. Mice in the control group (CG) were injected intramuscularly with 0.2 mL saline once a day for five consecutive days. Injections were made in the morning (at 8 to 9 AM) each day.

All mice were weighed each day using an electronic scale, and were raised as a group kept in cages (5 mice per cage), equipped with automatic water dispensers in a room maintained at 22–24°C and 30–50% relative humidity. The light cycle in the room provided 12 h light/day. Mice received a commercial diet (Lanzhou Taihua Feed Co. Ltd., China). Water was provided *ad libitum*. All procedures referring to animal treatment were approved by the Experimental Animal Care and Use Committee of Gansu Province, the People's Republic of China.

### Sample collections and measurements

Five mice from each group were sacrificed by cervical dislocation on days 0, 7, 10, 15, 20, and 30 after they were anesthetized by injecting 0.1 mg/kg xylazine intramuscularly. Bilateral ovaries were aseptically harvested. The weight of each ovary was recorded immediately on an electronic scale. The average value was calculated based on the left and right ovaries of each mouse. Blood samples were also collected at each sampling time-point as described above. Serum was separated and stored at –20°C.

### Detection of protein concentrations in ovarian tissues

Protein concentrations in ovarian tissues were detected using a BCA Protein Assay Kit (Yeze Biotechnology Co., Ltd., China) according to the kit protocol. Analytical sensitivities were 0.025 mg/mL.

### Western blots of ARID1A and PTEN, and FSHR proteins in mouse ovaries

To evaluate the expression levels of ARID1A and PTEN genes and proteins in mice ovaries, western blot was carried out according to our previous report (19). The polyclonal antibodies of rabbit anti-ARID1A antibody-C-terminal (Ab176395; 1:2000), anti-PTEN antibody (Ab170941, Abcam Trade Co., China; 1:2000), and anti-FSHR antibody (BOSTER Biological Technology Co. Ltd, China; 1:1000) were utilized. They were diluted and incubated at 4°C overnight, followed by 1 h incubation at room temperature with the appropriate secondary antibody (goat-anti mouse IgG; 1:20000). Anti-β-actin mouse monoclonal antibody (Ab8226, Abcam Trade Co.) was diluted at 1:8000 for sample loading control. Blots were further developed using a chemiluminescence reagent (SuperSignal West Pico, USA). The integrated optical density (IOD) of the scanned band images and relative level of each protein was determined using Quantity One software (Bio-Rad Company, USA). The relative concentrations of ARID1A and PTEN proteins are reported as the ratio between gray values of ARID1A and PTEN proteins divided by that of β-actin. A negative control was performed without a primary antibody. Assays were executed in triplicate.

### Real-time RT-PCR of FSHR mRNAs

#### Primer design

Primers specific for FSHR (GenBank accession number: NM-013523.3) were designed with Beacon Designer 7.0 software (Premier Biosoft International, USA) according to manufacturer's guidelines, and Primer-BLAST (NCBI-NIH, USA). Mouse GAPDH gene (GenBank accession number: NM-008084.2) was selected as the reference gene for normalizing expression levels of target genes. The primer sequences were as follows: FSHR, forward 5′-CGTCCTGATGAGCAAGTTTGG-3′ with 20 bp length, and reverse, 5′-TGGGCTGATTGACTTAGAGGG-3′; GAPDH, forward, 5′-CTTCAACAGCGACACTCACTCT-3′ and reverse, 5′-CCACCACCCTGTTGCTGTA-3′. Primers were synthesized by Beijing AoKeDingSheng Biotechnology Co. Ltd., China. The 100, 200, 300, and 500 nM concentrations were evaluated.

The constancy of GADPH was evaluated using geNorm. GAPDH level of CG on day 0 was used to normalize other gene expressions. Only those primer concentrations that showed dimmer-free reactions were used for the further analysis.

#### RNA extraction and cDNA synthesis

Total RNA was extracted from ovarian samples using the TRIzol reagent (Invitrogen, China), according to the manufacturer's instructions.

#### Fluorescence quantitative RT-PCR (qRT-PCR)

The expression levels of FSHR mRNAs were determined using qRT-PCR. Gene amplifications were performed using a SLAN thermocycler (Hongshi, China). Each 25-μL reaction volume in a 96-well plate comprised 4 μL of 50× diluted cDNA template and 1 μL of each primer pair at 10 μM. Plates were sealed with adhesive optical film (Promega, China) and, after an initial denaturation step of 15 min at 95°C, 44 cycles of amplification were performed according to the following thermocycling profiles: denaturation for 30 s at 95°C, annealing for 20 s at 60°C, and extension for 20 s at 72°C. Fluorescence data were acquired during the last step. A dissociation protocol with a gradient from 65 to 97°C was used to investigate the specificity of the qRT-PCR reaction and the presence of primer dimers. Gene expression levels were recorded as threshold cycle (C_T_) values that corresponded to the number of cycles at which the fluorescence signal can be detected above a threshold value, arbitrarily set to 0.3. GAPDH was used as an endogenous control. The relative amount of each mRNA was determined by the 2^–ΔΔ(Ct)^ method and normalized to the endogenous reference gene GAPDH, of CG on day 0. The samples were run in triplicate.

### Detection of serum concentrations of ARID1A and PTEN

Serum concentrations of ARID1A and PTEN were determined using the specific ELISA kit for mice (Shanghai Chenlie Biotech Co., Ltd., China) according to the kit protocol. Analytical sensitivities were 0.01 pg/mL. The intra- and inter-experimental coefficients of variation were less than 6%. The correlation coefficient of the standard curve was 0.9996. All samples were tested in duplicate in the same assay. The detailed operation steps are presented in our initial research (19).

### Statistical analysis

Statistical analysis was done using SPSS v. 21.0 (IBM, USA). For each group, all parameters (including ovarian cortex thickness, maximum longitudinal diameter, maximum transversal diameter, levels of ERβ and FSHR, serum E_2_, and FSH) were calculated based on the data of 5 mice in each subgroup. Data from each time-point were analyzed separately and are reported as means±SE. All variables complied with the assumptions for a one-way ANOVA. When significant differences were identified, supplementary Tukey's *post hoc* tests were performed to determine the pairwise differences. Pearson's correlation analysis was used to determine relationships between FRBI doses and other indexes in CG and the four FRBI groups on day 20. P<0.05 was considered significant.

## Results

### Expression levels of ARID1A and PTEN in ovaries

**Figure 1.**
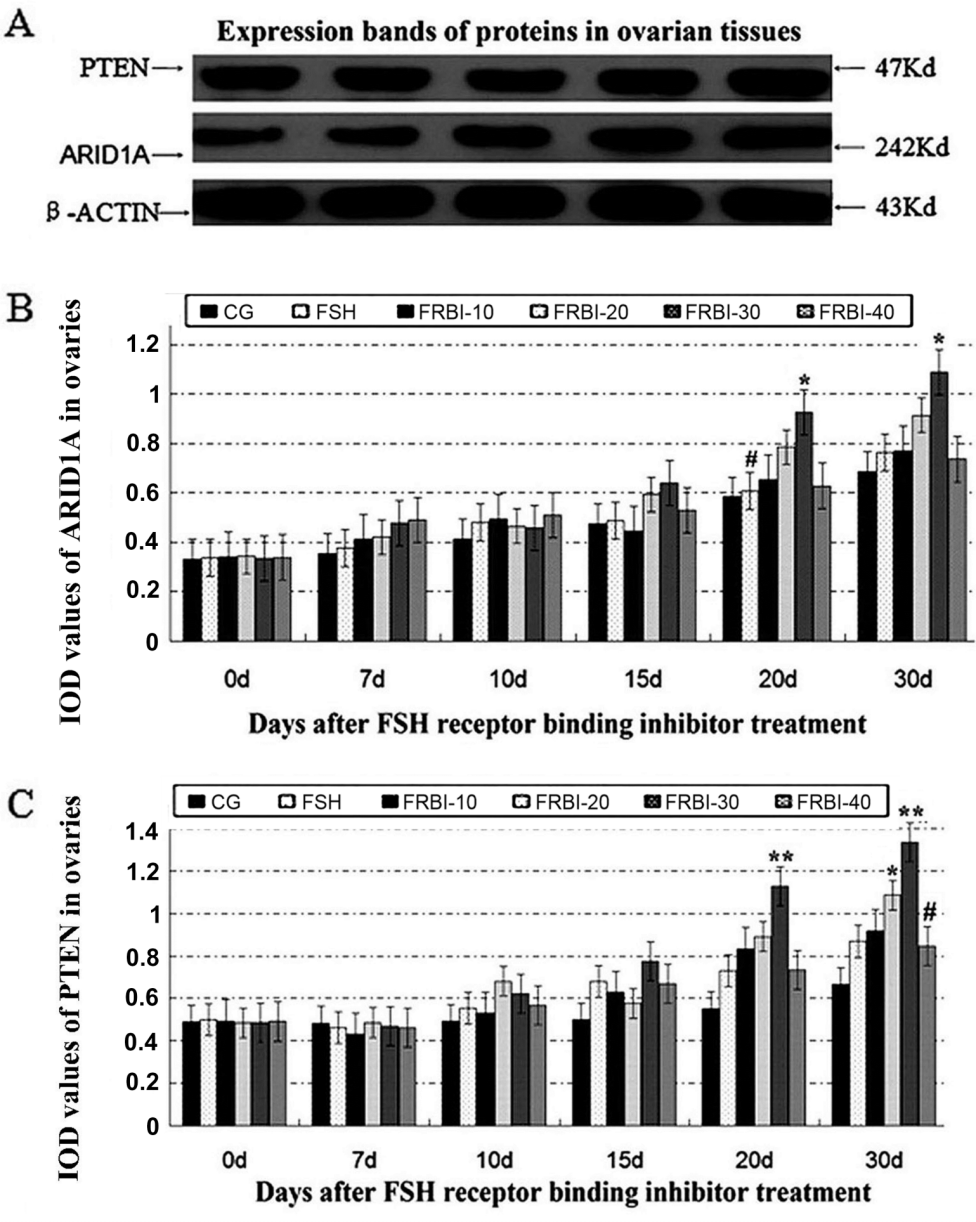
Expression levels of AT-rich interactive domain-containing protein 1A (ARID1A) and phosphatase and tensin homolog (PTEN) proteins in mouse ovaries. **A**, Western blotting results. **B** and **C**, ARID1A and PTEN proteins after different concentrations of FSH receptor binding inhibitor (FRBI-10 to -40). Data are reported as means±SE. *P<0.05, **P<0.01 compared to control group (CG); ^#^P<0.05 compared to follicle-stimulating hormone (FSH) group (ANOVA and Tukey's *post hoc* tests).

Western blotting assay showed clear bands of ARID1A and PTEN proteins (Figure 1A), indicating that they were expressed in mice ovaries at the different levels. Expression levels of ARID1A proteins were slightly increased after FRBI treatment (Figure 1B). ARID1A protein level of the FRBI-30 group was higher than that in CG on days 20 and 30 (P<0.05), and higher than in the FSH group on day 20 (P<0.05). These findings indicated that 30 mg/kg FRBI treatment could promote the expression level of ARID1A proteins in mouse ovaries.

As presented in Figure 1C, expression levels of PTEN proteins in the four FRBI groups were increased after treatment. The maximum increment was found in the FRBI-30 group. The PTEN level of the FRBI-30 group was higher than that in the CG on days 20 and 30 (P<0.01), and it was higher than the FRBI-40 group on day 30 (P<0.01). Meanwhile, PTEN level of the FRBI-20 group was higher than CG on day 30 (P<0.05). These findings indicated that 30 and 40 mg/kg FRBI treatment could enhance the expressions of PTEN proteins in the ovarian tissues of mice.

### Serum concentrations of ARID1A and PTEN

**Figure 2.**
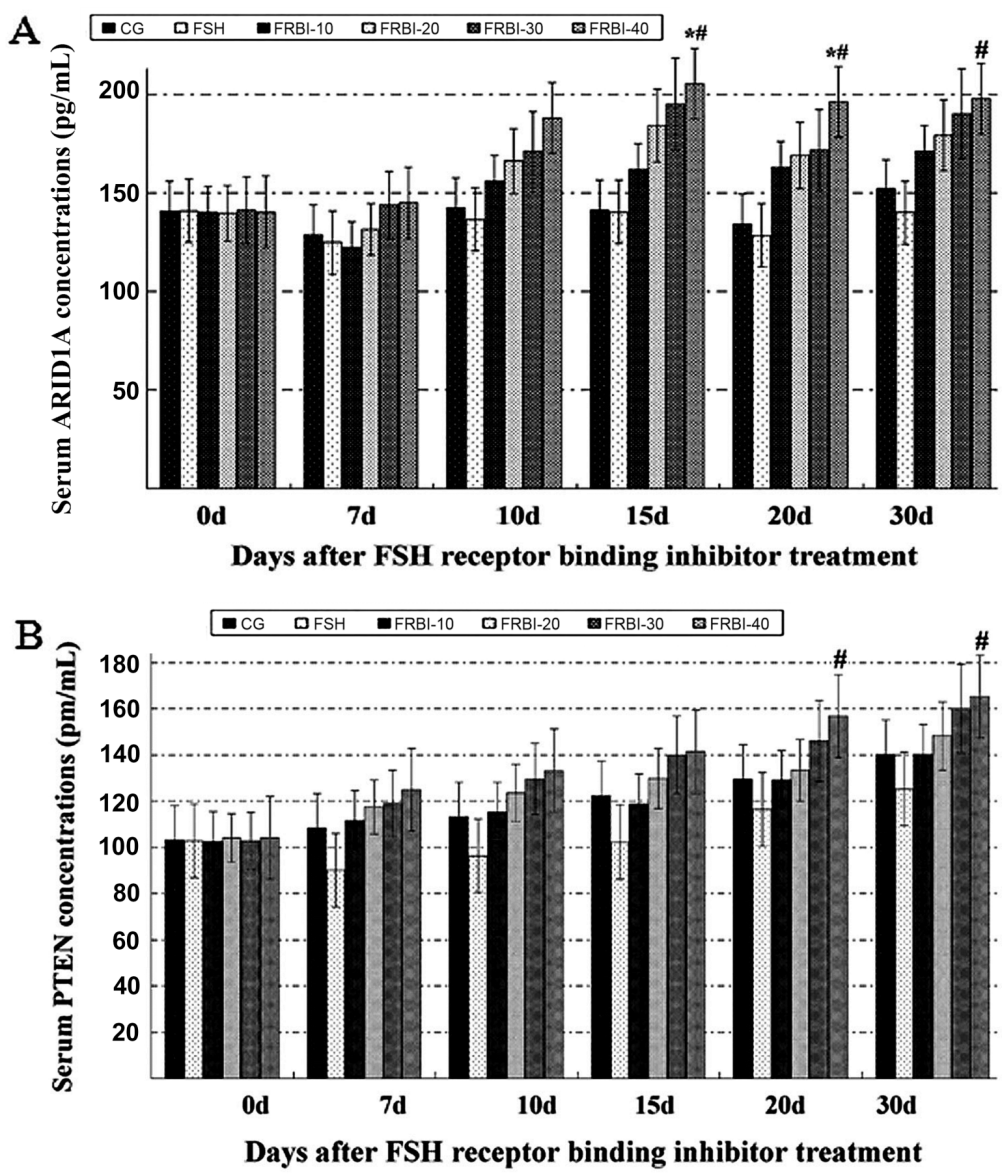
Serum AT-rich interactive domain-containing protein 1A (ARID1A) (**A**) and phosphatase and tensin homolog (PTEN) (**B**) concentrations after different concentrations of FSH receptor binding inhibitor (FRBI-10 to -40). Data are reported as means±SE. *P<0.05 compared to control group (CG); ^#^P<0.05 compared to follicle-stimulating hormone (FSH) group (ANOVA and Tukey's *post hoc* tests).

Serum ARID1A concentrations of FRBI-treated groups were increased along with the increase of FRBI doses (Figure 2A). ARID1A concentration of the FRBI-40 group was higher than that of CG and FSH groups on days 15, 20, and 30 (P<0.05). These results demonstrated that a high dose of FRBI improved production of ARID1A gene.

Serum PTEN concentrations changed similarly with concentrations of ARID1A (Figure 2B). PTEN concentrations of FRBI groups were gradually enhanced along with the increase of FRBI doses. PTEN concentration of FRBI-40 group was higher than that of FSH group on day 30 (P<0.05). However, there was no significant difference between the four FRBI groups and CG. These findings demonstrated that a high dose of FRBI (40 mg/kg) accelerated PTEN production and raised the serum concentrations of PTEN in mice.

### Expression levels of FSHR mRNA and proteins in ovaries

**Figure 3.**
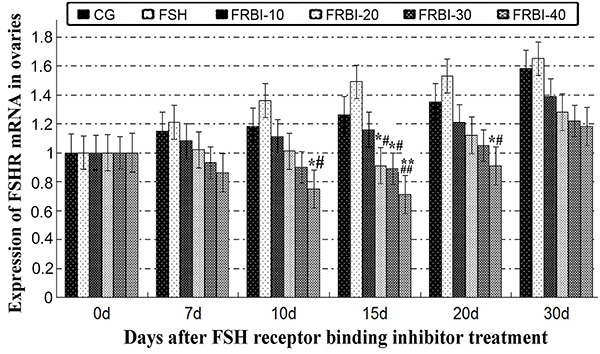
Expression levels of follicle-stimulating hormone cognate receptor (FSHR) mRNAs in mouse ovaries after different concentrations of FSH receptor binding inhibitor (FRBI-10 to -40). Data are reported as means±SE. *P<0.05, **P<0.01 compared to control group (CG), ^#^P<0.05, ^##^P<0.01 compared to follicle-stimulating hormone (FSH) group (ANOVA and Tukey's *post hoc* tests).

As shown in Figure 3, expression levels of FSHR mRNA of the four FRBI groups gradually declined in a dose-dependent manner, with a minimum value for the FRBI-40 group. FSHR mRNA level of the FRBI-40 group was less than that of CG and FSH groups on days 10 and 20 (P<0.05 or P<0.01). Meanwhile, FSHR mRNA levels of FRBI-20 and FRBI-30 groups were also lower than those of CG and FSH groups on day 15 (P<0.05 or P<0.01). The outcomes demonstrated that FRBI administration *in vivo* suppressed levels of FSHR mRNAs in mouse ovaries.

**Figure 4.**
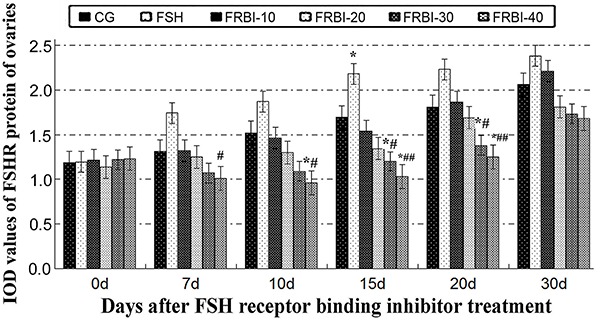
Integrated optical density (IOD) expression levels of follicle-stimulating hormone cognate receptor (FSHR) protein after different concentrations of FSH receptor binding inhibitor (FRBI-10 to -40) in mouse ovaries. Data are reported as means±SE. *P<0.05 compared to control group (CG); ^#^P<0.05, ^##^P<0.01 compared to follicle-stimulating hormone (FSH) group (ANOVA and Tukey's *post hoc* tests).

Expression levels of FSHR proteins were gradually increased in FSH-treated mice from day 7 after treatment (Figure 4). However, expression levels of FSHR proteins of all FRBI groups were dose-dependently reduced, with a maximum reduction in the FRBI-40 group. FSHR protein levels of FRBI-30 and FRBI-40 groups were lower than those of CG and FSH groups on days 15 and 20 (P<0.05 or P<0.01). These results revealed that a high dose of FRBI (30 or 40 mg/kg) could attenuate expression levels of FSHR proteins in mouse ovaries.

### Total protein concentrations of ovarian tissues

Total protein concentrations in the ovarian tissues of FRBI-treated groups were slightly decreased compared to CG on days 20 and 30 (data not shown). However, there was no significant difference among all groups. The results indicated that FRBI treatment did not affect the total protein concentrations in ovarian tissues of mice.

### Phosphorylation levels of FSHR proteins in serum and ovarian tissues

**Figure 5.**
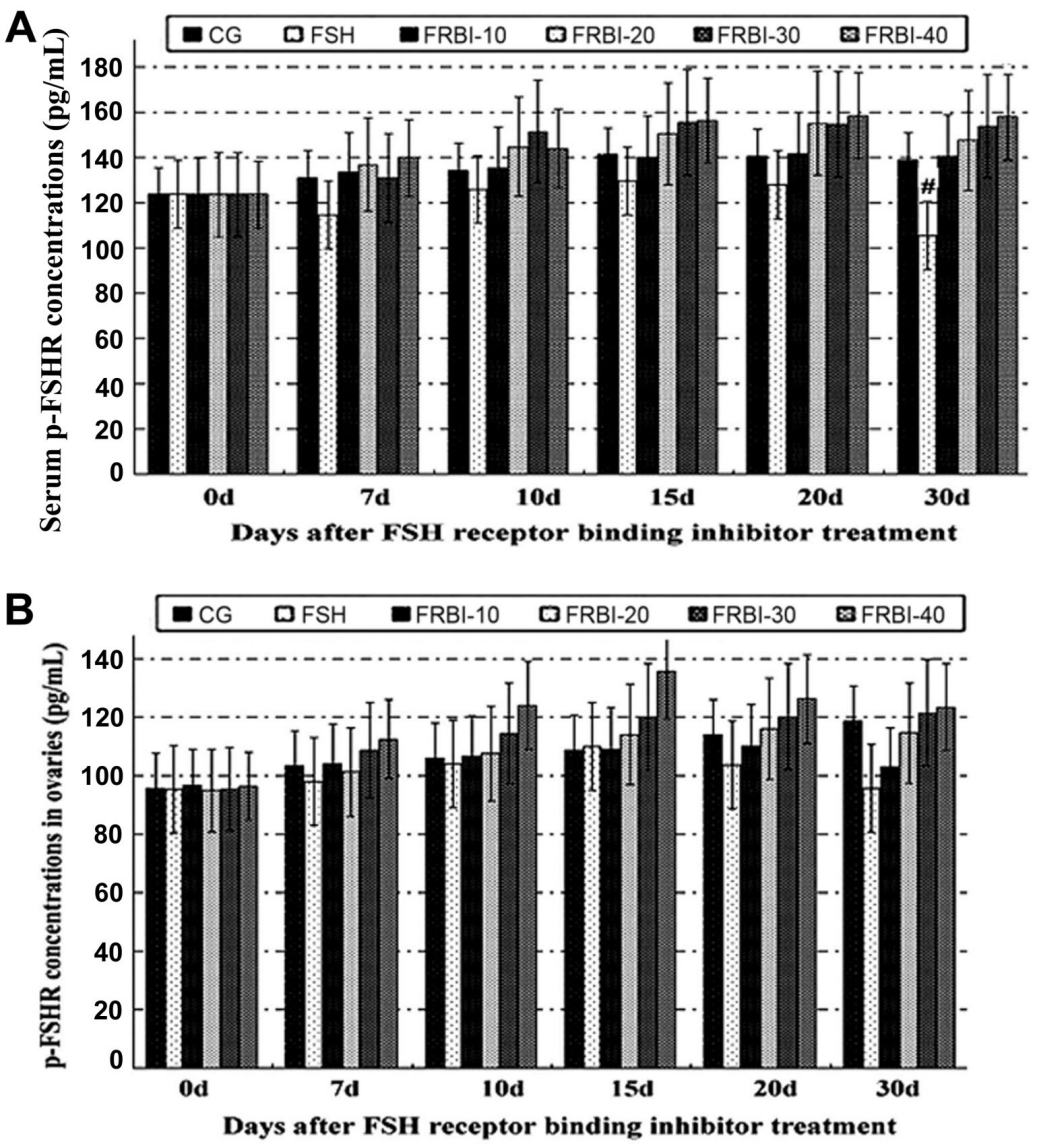
Phosphorylation level of follicle-stimulating hormone cognate receptor (FSHR) proteins in serum (**A**) and ovarian tissues (**B**) after different concentrations of FSH receptor binding inhibitor (FRBI-10 to -40). Data are reported as means±SE. At day 30, serum p-FHSR concentration of the FSH group was significantly less than that of the FRBI-40 group. However, there was no significant difference of serum p-FSHR concentrations between the other groups. (ANOVA and Tukey's *post hoc* tests).

Compared to CG, serum concentration of FSHR phosphorylation in the FSH group was slightly decreased in the experiment (Figure 5A). On day 30, concentration of FSHR phosphorylation in the FSH group was less than that of the FRBI-40 group (P<0.05). Contrarily, serum concentrations of FSHR phosphorylation in all FRBI groups were dose-dependently increased during the experiment. However, there was no significant difference between FRBI groups and CG. Ovarian concentrations of FSHR phosphorylation had no significant difference among groups during the whole experiment (Figure 5B). These results showed that FRBI treatment had no influence on the phosphorylation of FSHR protein in serum and ovarian tissues of mice.

### Pearson's correlation analyses

FRBI doses had significant positive correlations with levels of ARID1A and PTEN proteins (P<0.05; Table 1) and negative correlations with levels of FSHR mRNAs and proteins (P<0.05 or P<0.01). Additionally, ARID1A and PTEN had negative correlations with FSHR mRNA and protein levels (P<0.05 or P<0.01). These findings indicated that administration of FRBI had promoting effects on expression of ARID1A and PTEN in ovarian tissues.

**Table 1.** Pearson's correlation coefficients for all indexes on day 20.

Indexes	FRBI dose	TP	ARID1A	PTEN	FSHRm	FSHRp	p-FSHR
TP	-0.758						
ARID1A	0.944*	-0.928*					
PTEN	0.934*	-0.571	0.829				
FSHRm	-0.993**	0.827	-0.976**	-0.911*			
FSHRp	-0.937*	0.501	-0.788	-0.984**	0.898*		
p-FSHR	0.881*	-0.457	0.742	0.966**	-0.848	-0.968**	
IP3	-0.438	-0.105	-0.172	-0.415	0.341	0.553	-0.395

FRBI: FSH receptor binding inhibitor; TP: total proteins in ovarian tissue; ARID1A: AT-rich interactive domain-containing protein 1A; PTEN: phosphatase and tensin homolog; FSHRm: follicle-stimulating hormone cognate receptor mRNAs; FSHRp: FSHR proteins; p-FSHR: ovarian phosphorylated-FSHR; IP3: inositol trisphosphate. *P<0.05; **P<0.01.

## Discussion

Ovarian cancer (oophoroma), especially EOC, is a highly lethal gynecologic malignancy mostly because of delayed diagnosis (1). The early diagnosis of EOC is a key factor in improving the survival rate of patients. Thus, it is urgently necessary to search for novel diagnostic and prognostic biomarkers (10).

FSH acts via its cognate receptor (FSHR) expressed by granulosa cells in the follicles. FRBI blocked the binding of FSH to FSHR and altered FSH action. *In vivo* administration of FRBI impairs the proliferation of granulosa cells (11).

The identification of molecules and genes involved in the carcinogenesis of ovarian cancer has provided new insights into the molecular basis for anticancer therapy (20). ARID1A and PTEN are important tumor suppressors in various cancers. Their expression levels are correlated to carcinogenesis of ovarian and gynecologic cancers (6). There is little information regarding the influence of FRBI on ARID1A, PTEN, and FSHR associated with ovarian cancers (21,22) and no quantitative research has been reported about the correlation between ovarian carcinogenesis and levels of ARID1A and PTEN in humans and animals (1,23).

This study indicated that FRBI (30 or 40 mg/kg) increased the expression levels of both ARID1A and PTEN proteins in the ovaries. Our results were consistent with an earlier report (9). Therefore, FRBI, as a novel biomarker, could be utilized for early diagnosis and therapy of ovarian cancers (22). The actual effects of FRBI on anticancer activity and progression of ovarian cancers will need to be thoroughly investigated in the future (1).

Overexpression of FSHR increases proliferation in EOC cells. Inhibition of FSHR overexpression suppresses the tumorigenesis and progression of EOC (1). Moreover, whether the phosphorylation levels of FSHR proteins are implicated with the tumorigenesis is not well known (1,24). Our present results provided a solid foundation for decreasing the progression of ovarian cancer in patients through inhibiting FSHR overexpression in cancer tissues (25).

Protein phosphorylation plays an important role in the process of cell signal transduction, cell growth and development, and cancer mechanisms (26,27). Numerous studies in cancer cells and tissues resulted in identification of key differences from healthy tissues and cells at the protein level (28). Aberrant regulation of phosphorylation in signaling pathways contributes to carcinogenesis (29). Our findings preliminarily supported this opinion.

Our findings concerning serum concentrations of FSHR phosphorylation in FRBI groups and CG indicated that FRBI had no obvious influence on the phosphorylation level of FSHR in serum and ovarian tissues of mice. The main reasons may be that the dosage of FRBI was low and the age of experimental animals. Our results still need to be tested in other animals and humans.

## Conclusions

We concluded that FRBI treatment could increase the expression levels of ARID1A and PTEN proteins in mouse ovaries and serum, with a maximum increment in the FRBI-30 group (30 mg/kg). FRBI administration (30 or 40 mg/kg) decreased expression levels of FSHR mRNA and protein levels in mouse ovaries. FRBI doses had significant positive correlations to levels of ARID1A and PTEN proteins. However, FRBI had no influence on the total protein concentration and phosphorylation of FSHR. Our results provided new insights into the molecular foundation of FRBI and a potential novel medication for ovarian cancer (30).
